# Early diagnosis of dengue disease severity in a resource-limited Asian country

**DOI:** 10.1186/s12879-016-1849-8

**Published:** 2016-09-26

**Authors:** Philippe Cavailler, Arnaud Tarantola, Yee Sin Leo, Andrew A. Lover, Anne Rachline, Moniboth Duch, Rekol Huy, Ai Li Quake, Yuvatha Kdan, Veasna Duong, Jeremy L. Brett, Philippe Buchy

**Affiliations:** 1 Infectious Diseases Programme, Saw Swee Hock School of Public Health, National University of Singapore, Singapore, Singapore; 2 Epidemiology Unit, Institut Pasteur du Cambodge, Phnom Penh, Cambodia; 3 Institute of Infectious Disease and Epidemiology, Tan Tock Seng Hospital, Singapore, Singapore; 4 National Paediatric Hospital, Phnom Penh, Cambodia; 5 National Dengue Control Program (NDCP), National Center for Parasitological, Entomology and Malaria Control, Ministry of Health, Phnom Penh, Cambodia; 6 Virology Unit, Institut Pasteur in Cambodia, Phnom Penh, Cambodia; 7 Medical Affairs Department, Sanofi Pasteur, Singapore, Singapore; 8 Current address: Agence de Médecine Préventive, 13 Chemin du Levant, Ferney-Voltaire, 01210 France; 9 Current address: Global Health Group, University of California, San Francisco, USA; 10 Current address: Takeda Vaccines Pte Ltd., Singapore, Singapore; 11 Current address: GlaxoSmithKline vaccines, 150 Beach road, Singapore, Singapore

## Abstract

**Background:**

Dengue is endemic throughout Cambodia, a country faced with significant health and economic challenges. We undertook a clinical study at the National Paediatric Hospital in Phnom Penh to evaluate clinical diagnostic parameters for dengue and predictors of disease outcome.

**Methods:**

Between September 2011 and January 2013, all consecutive inpatients aged between 1 and 15 years and presenting with suspected dengue were enrolled. They were clinically assessed using both the 1997 and 2009 WHO dengue classifications. Specimens were collected upon admission and discharge and tested for dengue at Institut Pasteur in Cambodia.

**Results:**

A total of 701 patients were screened. Of these, 79 % were dengue-confirmed by laboratory testing, and 21 % tested dengue-negative. A positive tourniquet test, absence of upper respiratory symptoms, leukopenia, thrombocytopenia, and elevated liver transaminases were independent predictors for laboratory-confirmed dengue among the children. The presence of several warning signs on hospital admission was associated with a concurrent laboratory-confirmed diagnosis of severe disease outcome.

**Conclusions:**

The presence of two or more warning signs was associated with a concurrent laboratory-confirmed diagnosis of severe dengue at hospital admission. Thus, a cumulative score combining simple clinical parameters and first-line laboratory findings could be used to accurately predict dengue virus infection in pediatric populations, optimizing triage in settings with limited laboratory resources.

**Electronic supplementary material:**

The online version of this article (doi:10.1186/s12879-016-1849-8) contains supplementary material, which is available to authorized users.

## Background

Dengue is an acute febrile illness caused by one of four main dengue virus (DENV) serotypes (DENV-1, DENV-2, DENV-3 and DENV-4) and transmitted by *Aedes* spp. mosquitoes. Infection with DENV may remain asymptomatic or cause a spectrum of illnesses ranging from self-limiting influenza-like illness to life-threatening manifestations associated with plasma leakage and hemorrhage potentially leading to hypovolemic shock. DENV was first isolated in Cambodia in 1963. The disease is endemic, with peak epidemics observed each year during the rainy season.

Since 2000, the National Dengue Control Program of the Cambodian Ministry of Health (MOH) has reported an average of 103 cases per 100,000 population, with an annual case fatality rate ranging from 0.7 to 1.7 %. All four dengue serotypes have been found circulating in Cambodia with alternative predominance of serotypes DENV-2 and DENV-3. The DENV-1 represents from 5 to 20 % of all circulating viruses, depending on the year [1].

In resource-limited settings, the optimization of triage during dengue epidemics is crucial to avoid overwhelming health systems and to maximize the impact of available supportive treatments and resources. Therefore, in 2009, the World Health Organization (WHO) revised earlier guidelines to more accurately identify patients at risk of developing severe dengue and who may require hospitalization for closer clinical monitoring. Despite these guidelines, early diagnosis of dengue remains challenging for clinicians, as initial symptoms are generally indistinguishable from other endemic febrile illnesses in the tropics, and laboratory confirmation is not routinely available. Thus, there is currently no consensus on guidelines relying solely on clinical features to distinguish dengue from other febrile illnesses [2, 3]. While several prospective studies have described predictors for dengue diagnosis [4–6], none of these studies focused on the prediction of a concurrent laboratory-confirmed diagnosis of severe pediatric dengue at the time of hospital admission.

This study aims to describe the clinical spectrum of a large number of pediatric cases at a referral hospital in Phnom Penh, Cambodia, and to determine the clinical features predictive of laboratory-confirmed dengue infection. We also sought to determine, at hospital admission, the predictors of a concurrent laboratory-confirmed diagnosis of severe disease (Dengue Hemorrhagic Fever [DHF] as defined by the WHO 1997 guidelines [7] and Severe Dengue [SD] as defined by the WHO 2009 guidelines [8]).

## Methods

The study was conducted at the internal medicine and emergency wards of the National Paediatric Hospital in Phnom Penh. All children aged 1 to 15 years who were hospitalized between September 2011 and January 2013 and presented with acute onset of fever (temperature of > 37.5 °C on admission or history of fever less than 7 days) were screened for eligibility using both the 1997 and the 2009 WHO guidelines criteria (Additional file 1: Eligibility Form). All patients with suspected dengue were enrolled after informed written consent was obtained from the patients’ parents or guardians. Basic demographic, clinical and laboratory data were collected by trained clinicians at admission, using standardized Case Report Forms for all eligible patients (Additional file 2: CRF). Clinical information included the signs and symptoms listed in the 1997 and 2009 WHO case definitions. Basic laboratory data included the results of white blood cell and platelet counts, hematocrit and liver transaminases titers. The study was approved by the Cambodian National Ethics Committee for Health Research (approval number 123-NECHR, 22 August 2011).

Serum samples were collected at the time of admission during the acute febrile phase of dengue infection (“acute sample”) and at the time of hospital discharge (“convalescent sample”), ideally at least 7 days after the collection of the acute sample to allow interpretation of hemagglutination inhibition test results. Other details of this study have been reported previously [9].

Paired serum specimens were tested using an immunoglobulin M (IgM)-antibody capture enzyme-linked immunosorbent assay (MAC-ELISA) and a hemagglutination inhibition (HI) assay. Because of possible cross-reactivity, all specimens were systematically tested for anti-dengue virus and anti-Japanese encephalitis virus IgM using an in-house MAC-ELISA and a HI assay. The acute sample was tested for viral ribonucleic acid (RNA) by a serotype-specific quantitative real-time RT-PCR (qRT-PCR) method [10]. In addition, virus isolation was performed by inoculating sera into C6/36 *Aedes albopictus* and Vero E-6 cell cultures, and the virus serotype was identified using a direct fluorescent antibody assay of monoclonal antibodies [11]. Patients with negative qRT-PCR and inconclusive serological testing were tested for NS1 antigen using an ELISA capture commercial kit (Panbio Dengue Early ELISA, Panbio©, Sinnamon Park, Australia).

A case was considered positive for dengue when laboratory tests met one or more of the following criteria [8, 12]: 1) Dengue viral RNA detected by qRT-PCR or virus isolated after inoculation into cell cultures; 2) Seroconversion of DENV-specific IgM detected by MAC-ELISA in paired acute and convalescent samples; 3) Four-fold increase of HI antibody titer between acute and convalescent sera; 4) Dengue NS1 antigen detection.

All statistical analyses were conducted using SPSS version 20 (PNSW, Chicago, IL, USA) and Stata 12.1 (StataCorp, College Station, Texas, USA). We performed univariate analysis to identify the features associated with dengue positivity and severe disease. For the analyses on the predictors for dengue virus infection, we included all variables from the clinical assessment and the results of baseline laboratory parameters conducted upon hospital admission. We then assessed the association between the presence of warning signs on admission and the concomitant laboratory-confirmed diagnosis of severe disease (dengue hemorrhagic fever [DHF], dengue shock syndrome [DSS], or severe dengue [SD]). Mantel-Haenszel test were used for the comparisons of proportions (Fisher’s exact test if a cell had an expected frequency below five).

Multivariate logistic regression analysis was performed to identify variables independently associated with laboratory-positive dengue. The reference group included patients with a negative dengue laboratory confirmatory test. The initial logistic regression model included all covariates with a *p*-value of < 0.2 following univariate analysis. We then developed a score based on the results of the multivariate analysis; for each individual, each characteristic independently associated with dengue positivity was combined to provide an overall risk score. For each factor, categories with an odds ratio of ten or less relative to the baseline category were given a score of one, and categories with an odds ratio greater than ten were given a score of two. These scores were used to assess the ability to predict dengue infection via comparison of the sensitivity and specificity. Based on the procedures described in previous studies aiming to compare the performance of various clinical algorithms developed by WHO [13–15], the cut-off of each model was taken as the score that maximized the sum of the sensitivity and the specificity.

Receiver-Operator Characteristics (ROC) curves showing the Area Under the Curve (AUC) were generated to compare the overall ability of the different scores to predict dengue infection and concurrent laboratory-confirmed diagnosis of disease severity at hospital admission. Predicting accuracy was considered to be high if the AUC was > 0.9, moderate if in the range of 0.7 to 0.9, and low if between 0.5 and 0.7 [16].

## Results

From September 2011 to January 2013, a total of 1115 admitted cases presenting with acute febrile illness were evaluated, of which 701 (59 %) were clinically suspected of being dengue-positive as per the 1997 or 2009 WHO guidelines. Of these 701 suspected cases, 79 % (558/701) were laboratory-confirmed dengue cases, and 21 % (143/701) were laboratory-negative and classified as Other Febrile Illnesses (OFI). Of the dengue laboratory-positive cases, 531 (95.2 %) were confirmed by PCR (520 DENV-1; eight DENV-2; one DENV-3; two DENV-4); 27 had a negative PCR result and were confirmed by another test: eight by IgM seroconversion, eight by a four-fold increase of HI titer, and 11 by NS1. Among the 558 laboratory-confirmed dengue cases, gender was found to be evenly distributed (49.5 % female and 50.5 % male). The median duration of hospital stay was 3 days (Inter-Quartile Range [IQR] 2–4). Age varied between 1 and 15 years, with a median of 7 years (IQR 5–10), and 18.3 % were under 5 years of age. The median onset of fever prior to admission was 4 days (IQR 4–5) and did not differ between dengue and non-dengue febrile illnesses (Table 1).

**Table 1:** Demographics on admission of laboratory-positive and laboratory-negative patients, Dengue Surveillance Study (*N* = 701), National Paediatric Hospital, Phnom Penh, Sep. 2011–Jan. 2013

	DENV-Neg.	DENV-Pos.	*P*–value
	*n* = 143	*n* = 558	
Males (%)	50.0	50.5	>0.20
<5 years old (%)	24.5	16.7	0.09
Age: median [IQR]	7 [5–10]	7 [5–10]	>0.20
Onset of fever: median days [IQR]	4 [3–5]	4 [4–5]	0.29
Hospital stay: median days [IQR]	3 [3–4]	3 [2–4]	>0.20

**Fig. 1:**
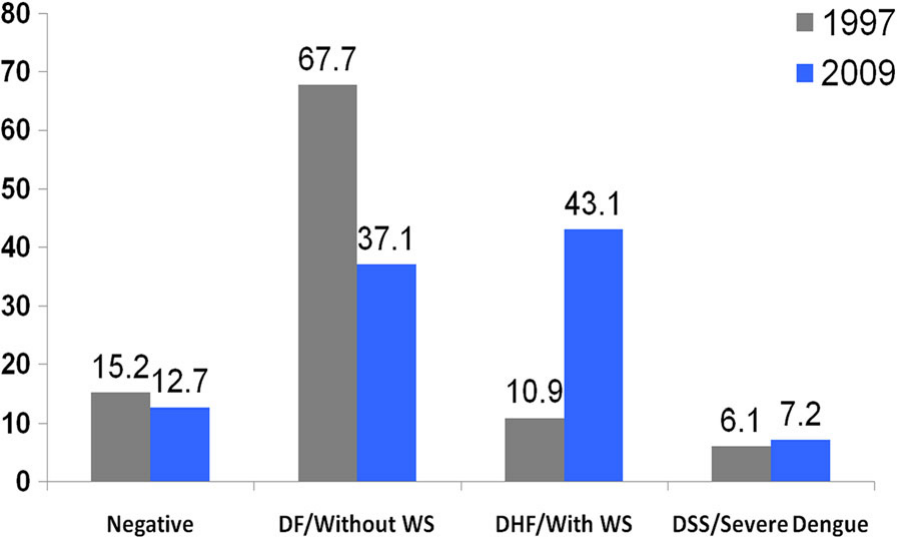
Classification of cases according to the criteria of the WHO 1997/2009 case definitions for dengue, National Paediatric Hospital, Phnom Penh, Sep. 2011–Jan. 2013

Using the 1997 WHO classification, 85 (15.2 %) of the dengue laboratory-confirmed cases would be classified as negative, 378 (67.7 %) as dengue fever (DF), 61 (10.9 %) as DHF, and 34 (6.1 %) as dengue shock syndrome (DSS). Using the 2009 WHO classification, 71 (12.7 %) of the dengue laboratory-confirmed cases would be classified as negative, 207 (37.1 %) as dengue without warning signs (WS), 240 (43.1 %) as dengue with WS, and 40 (7.2 %) as SD (Fig. 1).

### Clinical features of dengue infection

The clinical features of both laboratory-confirmed and negative patients were compared using multivariate analysis (Additional file 3: Supplementary Data Analyses). When the results of liver transaminases were included, we found the following five variables to be independently associated with laboratory-confirmed dengue: positive tourniquet test (odds ratio [OR] 2.17, 95 % confidence interval [CI] 1.32–3.59); absence of upper respiratory infection (URI) symptoms (OR 2.53, 95 % CI 1.47–4.37); platelet count < 100,000 cells per mm^3^ (OR 2.28, 95 % CI 1.32–6.37); leukocyte count < 4000 cells per mm^3^ (OR 3.60, 95 % CI 2.04–6.37); and liver transaminases > 60 IU/mL (either AST or ALT; OR 2.31, 95 % CI 1.42–3.75). When we excluded the results of ALT/AST testing, as these tests may not always be available in resource-limited settings, the following six variables were associated with laboratory-confirmed dengue infection: nausea or vomiting (OR 1.81, 95 % CI 1.10–2.98); absence of URI symptoms (OR 2.59, 95 % CI 1.63–4.14); positive tourniquet test (OR 2.22, 95 % CI 1.43–3.44); liver enlargement (OR 2.97, 95 % CI 1.31–6.74); platelet count < 100,000 cells per mm^3^ (OR 2.37, 95 % CI 1.48–3.82); and leukocyte count < 4000 cells per mm^3^ (OR 2.96, 95 % CI 1.82–4.83).

Risk scores were developed based on the cumulative total of the variables independently associated with laboratory confirmation of dengue from logistic regression models. For both scoring systems (with and without ALT/AST results), the prediction with the best sensitivity and specificity for laboratory-confirmed dengue integrated three criteria (Additional file 3). Two clinical scoring algorithms were developed based on the results of these analyses.

Table 2 shows the comparative performance of the two clinical algorithms in predicting laboratory-confirmed dengue. The two clinical algorithms (either with or without AST/ALT results) had a low specificity (57.4 and 65.7 %, respectively) but an acceptable level of sensitivity (84.2 and 75.7 %, respectively), with high positive predictive values. The ROC curves illustrate how these different models predict laboratory-confirmed dengue. The AUC was equal to 0.69 for the clinical algorithm without ALT/AST results, and to 0.71 for the algorithm with ALT/AST results.

**Table 2:** Performance of clinical algorithms in the prediction of dengue, National Paediatric Hospital, Phnom Penh, Sep. 2011–Jan. 2013

	Model A (with ALT/AST)	Model B (no ALT/AST)
Sensitivity (95 % C.I.)	75.7 (71.4–79.6)	84.2 (80.9–87.2)
Specificity (95 % C.I.)	65.7 (56.4–65.3)	57.4 (48.9–75.4)
Sensitivity + Specificity	141.4	141.6
Positive Predictive Value	89.7	88.3
Negative Predictive Value	40.8	48.8
AUC (95 % C.I.)	0.71 (0.65–0.77)	0.69 (0.63–0.75)

### Clinical features of DHF/DSS and severe dengue

We also explored the association between the presence of WS and the concurrent laboratory-confirmed diagnosis of DHF/DSS or SD at hospital admission. For both DHF/DSS and SD, the most frequent WS were clinical fluid accumulation (66 and 90 %, respectively), liver enlargement (55 and 65 %) and lethargy or restlessness (44 and 68 %). Table 3 describes the ability of the WS to predict laboratory-confirmed DHF/DSS or SD at hospital admission. Specificity was high for persistent vomiting (97 and 96 %, respectively), clinical fluid accumulation (96 and 91 %), and mucosal bleeding (92 and 90 %). Presentation of any WS was associated with a high sensitivity (92 %) but poor specificity in predicting severe outcomes. The presence of two or more WS at hospital admission yielded a sensitivity of 74 % and a specificity of 83 % in predicting a concurrent laboratory-confirmed diagnosis of DHF/DSS at hospital admission. The occurrence of three or more WS was associated with a sensitivity of 70 % and an increased specificity of 92 % for the prediction of a concurrent laboratory-confirmed diagnosis of SD at hospital admission.

**Table 3:** Performance of warning signs in the predictive diagnosis of dengue severe manifestations, National Paediatric Hospital, Phnom Penh, Sep. 2011–Jan. 2013

	DHF/DSS				Severe Dengue			
	Se	Sp	PPV^a^	NPV^b^	Se	Sp	PPV	NPV
Warning Signs								
Abdominal tenderness	43.2	77.0	43.2	86.8	40.0	74.6	10.9	94.1
Persistent vomiting	21.1	97.0	58.8	85.6	37.5	96.3	44.1	95.2
Clinical fluid accumulation	66.3	96.1	77.8	93.3	90.0	91.3	44.4	99.2
Mucosal bleeding	22.1	92.0	36.2	85.1	20.0	90.3	13.8	93.6
Lethargy/restlessness	44.2	83.3	35.3	87.9	60.0	81.6	20.2	96.3
Liver enlargement > 2 cm	55.8	82.0	39.0	90.0	65.0	78.7	19.1	96.7
Warning Signs Count								
• at least one	92.6	51.8	28.3	97.2	92.5	47.1	11.9	98.8
• at least two	73.7	82.9	47.0	93.9	80.0	77.4	21.5	98.0
• at least three	52.6	95.2	69.4	90.7	70.0	91.5	38.9	97.5
• at least four	22.1	98.3	72.4	86.0	42.5	97.7	58.6	95.7
• at least five	9.5	99.4	75.0	84.2	22.5	99.4	75.0	94.3
• All six are present	2.1	100.0	100.0	83.2	7.2	100.0	100.0	93.2

^a^ *PPV* positive predictive value

^b^ *NPV* negative predictive value

**Fig. 2:**
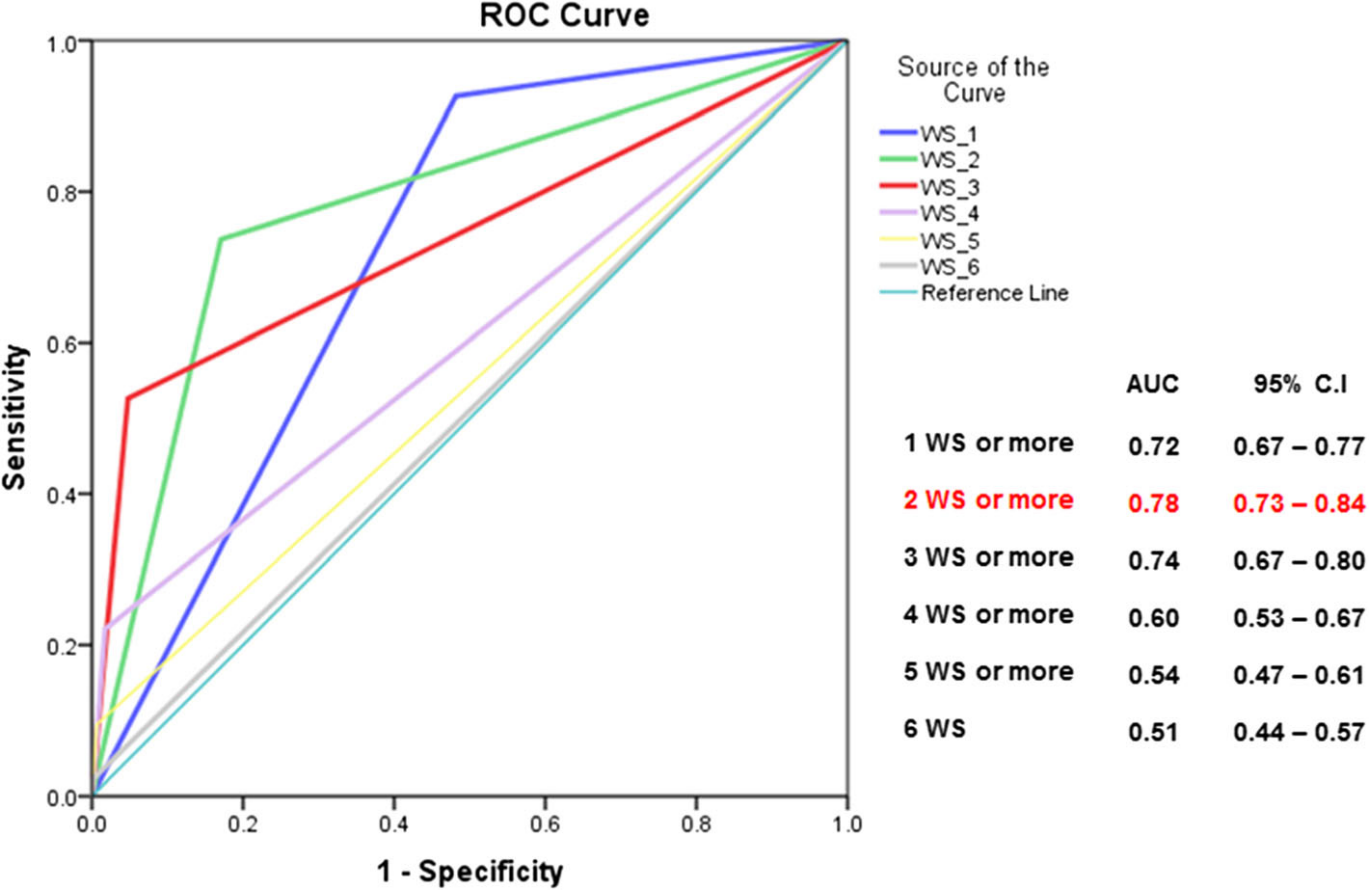
Receiver operator characteristics (ROC) curves comparing the predictive accuracy for DHF, of various models integrating one or several warning signs and presenting the values of the Area Under Curve (AUC) coefficients. National Paediatric Hospital, Phnom Penh, Sep 2011–Jan. 2013

**Fig. 3:**
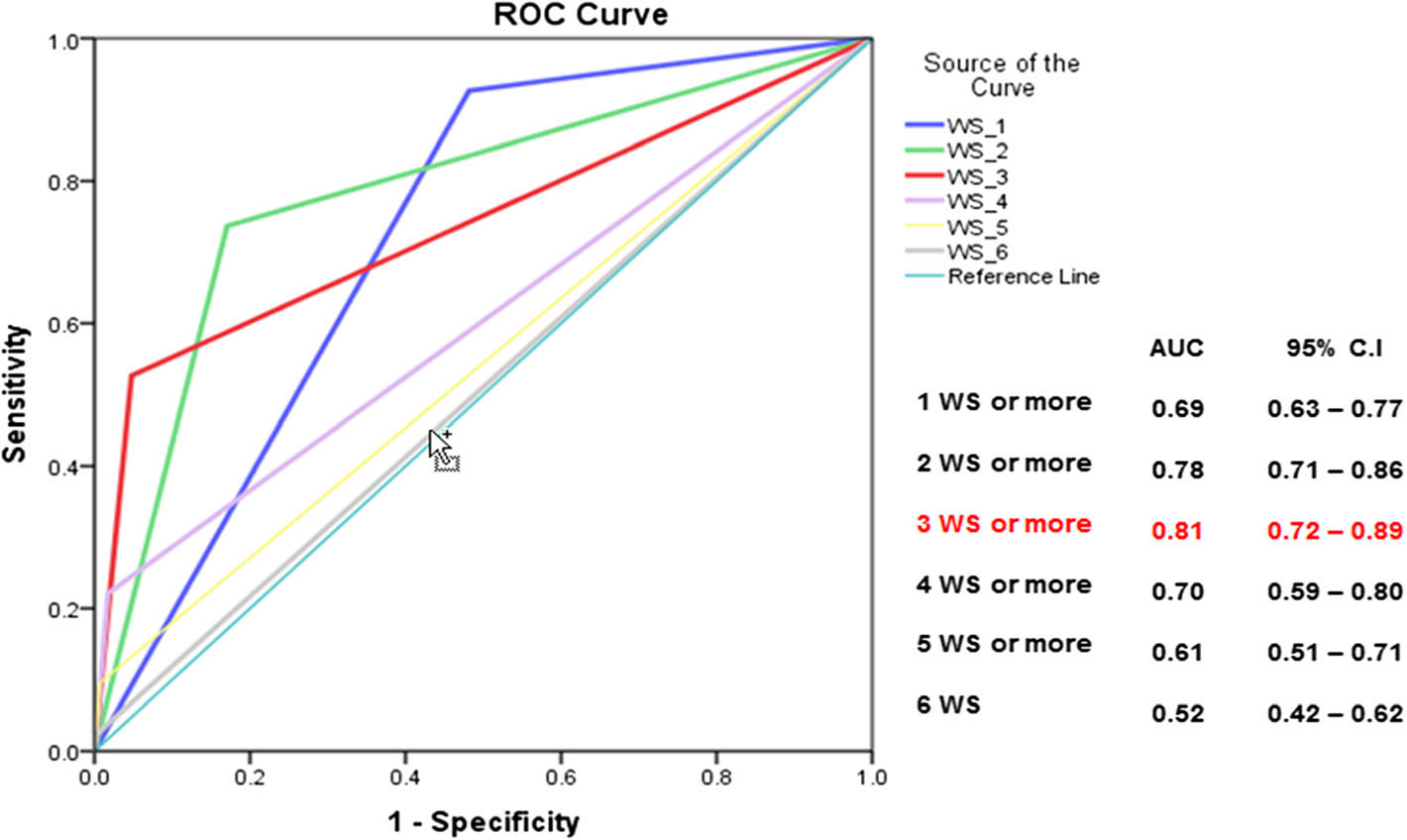
Received-operator characteristics (ROC) curves comparing the predictive accuracy for Severe Dengue of various models integrating one or several warning signs, and presenting the values of the Area Under Curve (AUC) coefficients. National Paediatric Hospital, Phnom Penh, Sep. 2011–Jan. 2013

The ROC curves (Figs. 2 and 3) illustrate the accuracy of these various models in predicting concurrent diagnosis of laboratory-confirmed DHF/DSS or SD at hospital admission, based on the total number of WS included in each model. For DHF/DSS, the highest predictive value was obtained when patients presented two or more WS upon admission (AUC = 0.78). For SD, predictive value was maximized when patients presented with at least three WS at admission (AUC = 0.81).

## Discussion

We conducted a 16-month clinical study of pediatric inpatients in a single referral hospital in Phnom Penh, Cambodia. While all 701 children enrolled in the study had clinical suspicion of dengue on admission, dengue was biologically confirmed in only 558 (79.6 %). This enabled us to compare children with dengue and those with non-dengue febrile illnesses. This information is particularly useful in resource-limited settings where dengue diagnostic tests are not readily available.

The study period, from September 2011 to January 2013, covered two seasons of dengue epidemics. Although all four serotypes were circulating during this period, the predominant serotype was DENV-1 (98 %) in 2012–2013, which is consistent with data from the National Surveillance System (Institut Pasteur du Cambodge, personal communication).

Overall, at hospital admission, 17 % of patients would be clinically classified as DHF or DSS according to the 1997 WHO case definition criteria, while 7.2 % would be classified as SD based on the 2009 WHO case definition. This low proportion of patients presenting with severe outcomes is congruent with the low case fatality rate (0.7 %) observed in our hospital cohort and might reflect the quality of case management. In Cambodia, patients die traditionally at home, and family members usually request that moribund relatives be discharged. For this reason, we conducted a post-discharge assessment in order to validate the vital status of a random sample of 20 study participants (from a total of 40 patients diagnosed with a severe form of dengue at admission) who resided outside Phnom Penh and presented with either DDS or SD at hospital admission. The heads of the villages confirmed that all 20 children were still alive at the time of the interview. The low case fatality rate observed in the study is encouraging. It is in line with the MOH’s 2012–2013 National Strategic Plan, in which the National Dengue Control Program trained hospital teams to rationalize hospital care and improve case management of severe manifestations of dengue.

Similar to previous studies [17], we found that clinical algorithms had an acceptable sensitivity for the detection of dengue (76 to 81 %) but quite low specificity (57 to 66 %) in the epidemiological context of this study in Cambodia, where other pathogens mimicking the clinical presentation of DENV are present. Therefore, one important study objective was using clinical parameters to develop new clinical algorithms that better predict dengue. We found that a positive tourniquet test, leukopenia, thrombocytopenia and elevated transaminases were independent predictors of dengue infection; these findings are congruent with the results of other studies [5, 18, 19]. The presence of elevated liver enzymes was frequently observed in the early stages of both DF and DHF [20, 21]. We also found that the presence of URI symptoms was useful in ruling out dengue, as the absence of this criterion is highly sensitive (82.4 %) for the presence of dengue in this cohort. The inclusion of URI symptoms in our clinical algorithm is essential, since it helps discriminate between dengue and viral respiratory infections; this finding is important in the daily clinical setting and has already been highlighted by other authors [3, 4, 22].

Using the 2009 WHO classification, the presence of any WS at hospital admission leads to a concurrent laboratory-confirmed diagnosis of a severe form of dengue. WS are intended to help triage patients at risk of developing severe outcomes and needing hospital admission in order to initiate early management and intensive monitoring immediately upon admission [23], and the utility of this approach has been confirmed by recent studies conducted in Sri Lanka [24] and Singapore [25]. In Cambodia, we found that any one of the WS was associated with a high sensitivity (93 %) but a low specificity (47 %) in predicting concomitant laboratory-confirmed diagnosis of a severe form of dengue. In resource-limited settings, this might put additional pressure on an already overburdened healthcare system, especially during annual dengue epidemics. Thus, the presence of several WS would improve the specificity (up to 91 % with at least three WS present) in predicting severe disease at the expense of sensitivity and might be an appropriate strategy in countries like Cambodia.

This study adds further evidence for the usefulness of WS. The absence of any WS at hospital admission is strongly associated with non-severe dengue, even in the absence of laboratory confirmation of dengue. Such cases are routinely managed at the outpatient department. The presence of a single WS at admission was found to be insufficiently specific to predict a concurrent laboratory-confirmed diagnosis of a severe outcome of dengue. Therefore, our results strongly suggest that dengue-suspected cases with two or more WS should be admitted, since this predicts severe dengue with a sensitivity of 74 % and a specificity of 83 %.

This study has some limitations. First, the data were collected among inpatients at a referral pediatric hospital in Phnom Penh, whose patient population may be biased towards patients with more severe diseases than those of hospitals at peripheral health center level. Thus, these findings might not describe the full spectrum of dengue in Cambodia and, hence, might not be fully representative of areas with different dengue transmission patterns or serotypes, or where patients seek medical care later in the course of illness. Because of limited resources, it was not possible to run additional biological tests to investigate the etiologies of the other febrile illnesses. Further efforts should be made to validate the findings in other epidemiological contexts. Second, there were incomplete data for certain variables (i.e. results of the tourniquet tests and AST/ALT), although we have no reason to suspect that this introduced a bias. Third, because of limited resources, we were unable to test the 482 patients who did not fulfill the WHO criteria for dengue on admission and who represented a significant number of the patients with fever (40 %). It is likely we missed some dengue cases by relying solely on the WHO criteria. The median duration of hospitalization was short (3 days), and a significant number of patients did not have a convalescent sera collected. This could have caused misclassification of non-lab confirmed cases. DENV-1 was the predominant serotype identified during the study period, and it was not possible to explore the effects of other dengue serotypes; this might affect the generalizability of our results, which remain context-specific. Finally we analyzed clinical data at hospital admission, and because of sample size limitation, we were unable to analyze the predictors for the progression to severe disease following hospital admission. Finally, the limited number of recruited cases limits the possibility to conduct further stratified analysis by dengue serotypes or time periods. The complex interaction between climatic factors and the risk of dengue infection in this setting has already been highlighted [9].

Considering these limitations, and although dengue presentation can vary, the strength of this study is that it directly relates to the most severe aspects of dengue, which can cause not only morbidity but also mortality. Furthermore, our study took place over two seasons in a hospital receiving cases referred from many areas in the country. The inclusion criteria and study monitoring were rigorously applied; all patients were systematically and thoroughly assessed, and testing was performed in an internationally recognized laboratory. Finally, there were few missing data, and we have no reason to believe that missing data were not randomly distributed across the study records. The study also reinforces the need to educate clinicians on key parameters for triage and case management in a context where laboratory diagnosis may not be easily available.

## Conclusions

We have described the clinical features of dengue among children in a referral hospital in Phnom Penh, Cambodia. Our study indicates that the addition of simple clinical parameters and first-line laboratory findings could be better used to predict concurrent laboratory-confirmed diagnosis of a severe form of dengue at hospital admission. While our findings remain context-specific, we demonstrate that the inclusion of multiple warning signs more accurately predicts a concomitant laboratory-confirmed diagnosis of severe disease at hospital admission, thereby optimizing triage in settings with limited laboratory resources. While some improvements can be achieved with these clinical parameters, access to dengue rapid diagnostic tests and basic laboratory testing should also be prioritized in resource-limited settings.

## Additional files

Additional file 1: Eligibility Form. (PDF 111 kb) Additional file 2: Case Report Form. (PDF 260 kb) Additional file 3: Supplementary Data Analyses. (DOC 190 kb)

## References

[1] Huy R, Buchy P, Conan A, Ngan C, Ong S, Ali R, Duong V, Yit S, Ung S, Te V, Chroeung N, Phaektra NC, Uok V, Vong S (2010). National dengue surveillance in Cambodia 1980–2008: epidemiological and virological trends and the impact of vector control. Bull World Health Organ.

[2] Farrar J, Focks D, Gubler D, Barrera R, Guzman MG, Simmons C, Kalayanarooj S, Lum L, McCall PJ, Lloyd L, Horstick O, Dayal-Drager R, Nathan MB, Kroeger A (2007). Towards a global dengue research agenda. WHO/TDR Dengue Scientific Working Group. Trop Med Int Health.

[3] Ramos MM, Tomashek KM, Arguello DF, Luxemburger C, Quinones L, Lang J, Munoz-Jordan JL (2009). Early features of dengue infection in Puerto Rico. Trans R Soc Trop Med.

[4] Gregory CJ, Santiago LM, Arguello DF, Hunsperger E, Tomashek KM (2010). Clinical and laboratory features that differentiate dengue from other febrile illnesses in an endemic area–Puerto Rico, 2007–2008. Am J Trop Med Hyg.

[5] Kalayanarooj S, Vaughn DW, Nimmannitya S, Green S, Suntayakorn S, Kunentrasai N, Viramitrachai W, Ratanachu-eke S, Kiatpolpoj S, Innis BL, Rothman AL, Nisalak A, Ennis FA (1997). Early clinical and laboratory indicators of acute dengue illness. J Inf Dis.

[6] Tuan NM, Nhan HT, Chau NVV, Hung NT, Tuan HM, Tram TV, Ha NL, Loi P, Quang HK, Kien DTH, Hubbards S, Chau TNBC, Wills B, Wolbers M, Simmons CP (2015). Sensitivity and specificity of a novel classifier for the early diagnostic of dengue. PLoS Negl Trop Dis.

[7] WHO (2007). Dengue haemorrhagic fever; guidelines for diagnosis, prevention, treatment and control.

[8] WHO (2009). Dengue guidelines for diagnosis, treatment, prevention and control. New edition.

[9] Lover AA, Buchy P, Rachline A, Moniboth D, Huy R, Meng CY, Leo YS, Yuvatha K, Sophal U, Chantha N, Y B, Duong V, Goyet S, Brett JL, Tarantola A, Cavailler P (2014). Spatial epidemiology and climatic predictors of paediatric dengue infections captured via sentinel site surveillance, Phnom Penh, Cambodia 2011–2012. BMC Public Health.

[10] Hue KD, Tuan TV, Thi HT, Bich CT, Anh HH (2011). Validation of an internally controlled one-step real-time multiplex RT-PCR assay for the detection and quantification of dengue virus RNA in plasma. J Virol Methods.

[11] Gubler DJ, Yunker C (1986). Application of serotype-specific monoclonal antibodies or identification of dengue viruses. Arboviruses and arthropod cells in vitro.

[12] Peeling RW, Artsob H, Pelegrino JL, Buchy P, Cardosa MJ, Devi S, Enria DA, Farrar J, Gubler DJ, Guzman MG, Halstead SB, Hunsperger E, Kliks S, Margolis HS, Nathanson CM, Nguyen VC, Rizzo N, Vázquez S, Yoksan S (2010). Nat Rev Microbiol.

[13] Vuylsteke B, Laga M, Alary M, Gerniers MM, Lebughe JP, Nzila N, Behets F, Van Dyck E, Piot P (1993). Clinical algorithms for the screening of women for gonococcal and chlamydial infection: evaluation of pregnant women and prostitute in Zaire. Clin Infect Dis.

[14] Mayaud P, Uledi E, Cornelissen J, ka-Gina G, Todd J, Rwakatare M, West B, Kopwe L, Manoko D, Grosskurth H, Hayes R, Mabey D (1998). Risk scores to detect cervical infections in urban antenatal clinic attenders in Mwanza, Tanzania. Sex Transm Inf.

[15] Cornier N, Petrova E, Cavailler P, Dentcheva R, Terris-Preshold F, Janin A, Ninet B, Anguenot JL, Vassilakos P, Gerbase A, Mayaud P (2010). Optimising the management of vaginal discharge in Bulgaria: cost effectiveness of four clinical algorithms with risk assessment. Sex Transm Inf.

[16] Pepe MS (2003). The statistical evaluation of medical tests for classification and prediction.

[17] Low JGH, Ong A, Tan LK, Chaterji S, Chow A, Lim WY, Lee KW, Chua R, Chua CR, Tan SWS, Cheung YB, Hibberd ML, Vasudevan SG, Ng LC, Leo YS, Ooi EE (2011). The early clinical features of dengue in adults: challenges for early clinical diagnosis. PLoS Negl Trop Dis.

[18] Phuong CXT, Nhan NT, Wills B, Kneen R, Ha NTT, Mai TTT, Huynh TTT, Lien DTK, Solomon T, Simpson JA, White NJ, Farrar J, the Dong Nai Paediatric Hospital Study Group (2002). Evaluation of the World Health Organization standard tourniquet test and a modified tourniquet test in the diagnostic of dengue infection in Vietnam. Trop Med Int Health.

[19] Hammond SN, Balmaseda A, Perez L, Tellez Y, Saborio SI, Mercado JC, Videa E, Rodriguez Y, Perez MA, Cuadra R, Solano S, Rocha J, Idiaquez W, Gonzalez A, Harris E (2005). Differemces in dengue severity in infants, children and adults in a 3-year hospital-based study in Nicaragua. Am J Trop Med Hyg.

[20] Wilder-Smith A, Earnest A, Paton N (2004). Use of simple laboratory features to distinguish the early stage of severe acute respiratory syndrome from dengue fever. Clin Infect Dis.

[21] Lee LK, Gan VC, Lee VL, Tan AS, Leo YS, Lye DC (2012). Clinical relevance and discriminatory value of elevated liver amino transferase levels for dengue severity. PLoS Negl Trop Dis.

[22] Phuong CXT, Nhan NT, Kneen R, Thuy PTT, Thien CV, Nga NTT, Thuy TT, Solomon T, Stepniewska K, Wills B, the Dong Nai Study Group (2004). Clinical diagnosis and assessment of severity of confirmed dengue infections in Vietnamese children: is the World Health Organization classification system helpful?. Am J Trop Med Hyg.

[23] Leo YS, Gan VC, Ng EL, Hao Y, Ng LC, Pok KY, Dimatatac F, Go CJ, Lye DC (2013). Utility of warning signs in guiding admission and predicting severe disease in adult dengue. BMC Inf Dis.

[24] Jayaratne SD, Atukorale V, Gomes L, Chang T, Wijesinghe T, Fernado S, Ogg GS, Malavige GN (2012). Evaluation of the WHO revised criteria for classification of clinical disease severity in acute dengue infection. BMC Res Note.

[25] Thein TL, Gan VC, Lye DC, Yung CF, Leo YS (2013). Utilities and limitations of the World Health Organization 2009 warning signs for adult dengue severity. PLoS Negl Trop Dis.

