# Supplementary material for: Early diagnosis of dengue disease severity in a resource-limited Asian country
Source: BMC Infect Dis. 2016 Sep 26;16:512. doi: 10.1186/s12879-016-1849-8 (PMC5036306; doi:10.1186/s12879-016-1849-8)
Supplement: Additional file 1: — Eligibility Form. (PDF 111 kb) [file 12879_2016_1849_MOESM1_ESM.pdf]

Eligibility Form – Dengue Study *(Ve. 120426)*

Name Patient .... **Study Nurse** ....

Age I__I__I **Ward** ❑ I ❑ E

Date of Admission |__|__| |__|__| |__|__|__|__| **Time of Admission** |__|__| |__|__|

Temperature |__|__|.|__| **Fever duration prior admission: I__I__I days**

WHO Case Definition 1997

**Fever lasting 2-7 days**	❑ No	❑ Yes	
**Criteria1: Headache**	❑ No	❑ Yes	❑ Don't know *(cannot express)*
**Criteria 2: Retro orbital pain**	❑ No	❑ Yes	❑ Don't know *(cannot express)*
**Criteria 3: Arthralgia**	❑ No	❑ Yes	❑ Don't know *(cannot express)*
**Criteria 4: Myalgia**	❑ No	❑ Yes	❑ Don't know *(cannot express)*
**Criteria 5: Rash**	❑ No	❑ Yes	
**Criteria 6: Hemorrhagic Manifestations**	❑ No	❑ Yes	
**Criteria 7: Leukopenia**	❑ No	❑ Yes *($< 5 \times 10^9$ / L)*	
**Eligibility WHO 1997**	❑ No	❑ Yes *(fever 2-7 days, plus at least 2 criteria)*	

WHO Case Definition 2009

**Fever lasting 2–7 days**	❑ No	❑ Yes	
**Criteria1: Nausea, vomiting**	❑ No	❑ Yes	
**Criteria 2: Rash**	❑ No	❑ Yes	
**Criteria 3: Aches and pains**	❑ No	❑ Yes	
**Criteria 4: Tourniquet test**	❑ Neg.	❑ Pos.	❑ Not performed
**Criteria 5: Leukopenia**	❑ No	❑ Yes *($< 5 \times 10^9$ / L)*	
**Criteria 6: Warning signs**	❑ No	❑ Yes *(specify which ones below)*	

|__| **Abdominal pain, tenderness** |__| **Persistent vomiting**

|__| **Clinical fluid accumulation** *(ascites, pleural effusion …)*

|__| **Lethargy, restlessness** |__| **Liver enlargement > 2 cm**

|__| **Mucosal bleed** *(bleeding nose, bleeding gums…)*

|__| **Increased haematocrit and / or rapid decrease of platelets**

Eligibility WHO 2009 ❑ No ❑ Yes *(fever lasting 2-7 days, and at least 2 criteria)*

Outcome: ❑ **Not Eligible** ❑ **Eligible but Refusal** ❑ **Eligible: ID I__I I__I__I__I**

Validation: Dr ....

Signature

Eligibility Form – Dengue Study *(Ve. 120426)*

REFUSAL BOX

If parents refuse the inclusion of the child, fill the eligibility card, and report below the reason for the refusal

I__I My child is too young

I__I My child is too sick

I__I Other reason, *please specify:* ....
