# Supplementary material for: Early diagnosis of dengue disease severity in a resource-limited Asian country
Source: BMC Infect Dis. 2016 Sep 26;16:512. doi: 10.1186/s12879-016-1849-8 (PMC5036306; doi:10.1186/s12879-016-1849-8)
Supplement: Additional file 2: — Case Report Form. (PDF 260 kb) [file 12879_2016_1849_MOESM2_ESM.pdf]

CASE REPORT FORM

(April 2012)

Surveillance of Paediatric Dengue

National Paediatric Hospital, Phnom Penh
Kingdom of Cambodia

Study contact:

I am confident that the information supplied in this case record form is complete and accurate data. I confirm that the study was conducted in accordance with the protocol and any protocol amendments and that written informed consent was obtained prior to the study.

Name of the Investigator:

Investigator's Signature:

Date of signature: □□ / □□ / □□□□

Subject Initials: I__I__I__I

Subject ID Number I__I__I__I__I

I. Informed Consent

Written informed consent must be given by the parents before inclusion in this study.

តើពត៌មាននៃការយល់ព្រមបានផ្តល់ឱ្យឬទេ?/Has informed consent been given? ❑$_0$ No ❑$_1$ Yes

If NO, do NOT proceed.

II. ការសំរាកព្យាបាល/Admission

ថ្ងៃចូលសំរាកព្យាបាល/Date ☐☐ / ☐☐ / ☐☐☐☐ ម៉ោង/Time ☐☐ / ☐☐

បន្ទប់/Ward ❑$_1$ Infectious Diseases ❑$_2$ Emergency Admission Number ______

Demographic Data

អាយុ/Age ☐☐Years or ☐☐ Months ភេទ/Sex ❑$_1$ ប្រុស/Male ❑$_2$ ស្រី/Female

Address: ____

Medical History

Previous dengue infection ❑$_0$ No ❑$_1$ Yes ❑$_9$ Don't know How recently? ☐☐ Months

Confirmed where? ____________________________________ or ☐☐Yrs ago

History of YF Vaccination ❑$_0$ No ❑$_1$ Yes ❑$_9$ Don't know

History of JE vaccination ❑$_0$ No ❑$_1$ Yes ❑$_9$ Don't know

Fever days prior to admission I___I___I Days

Infusion prior to admission ❑$_0$ No ❑$_1$ Yes ❑$_9$ Don't know

Symptoms on Admission (Day I)

Fever	❑$_0$ No	❑$_1$ Yes	History Fever (2-7 days)	❑$_0$ No	❑$_1$ Yes
Chills	❑$_0$ No	❑$_1$ Yes	Rash	❑$_0$ No	❑$_1$ Yes
Pallor or cool skin	❑$_0$ No	❑$_1$ Yes	Vomiting	❑$_0$ No	❑$_1$ Yes
Headache	❑$_0$ No	❑$_1$ Yes	Nausea	❑$_0$ No	❑$_1$ Yes
Retro-orbital pain	❑$_0$ No	❑$_1$ Yes	Anorexia	❑$_0$ No	❑$_1$ Yes
Muscle pain	❑$_0$ No	❑$_1$ Yes	Abdominal pain	❑$_0$ No	❑$_1$ Yes
Joint pain	❑$_0$ No	❑$_1$ Yes	Abdominal distension	❑$_0$ No	❑$_1$ Yes
Backache	❑$_0$ No	❑$_1$ Yes	Diarrhoea (> 3 stools/day)	❑$_0$ No	❑$_1$ Yes
Convulsion or coma	❑$_0$ No	❑$_1$ Yes	Jaundice	❑$_0$ No	❑$_1$ Yes
Restless	❑$_0$ No	❑$_1$ Yes	Sore throat	❑$_0$ No	❑$_1$ Yes
Lethargy	❑$_0$ No	❑$_1$ Yes	Cough	❑$_0$ No	❑$_1$ Yes
Confusion	❑$_0$ No	❑$_1$ Yes	Breathlessness	❑$_0$ No	❑$_1$ Yes

Other (specify):____

Subjects Initials I__I__I__I Subject ID Number I__I__I__I__I

Clinical Examination on Admission (Day I)

លក្ខខ័ណ្ឌទូទៅ/General condition	❑$_1$ មើលទៅល្អ/Looks Well	❑$_2$ មើលទៅមិនល្អ/Looks Unwell		
ទំងន់/Weight	☐☐.☐ kg		កំពស់/Height	☐☐☐cm
សីតុណ្ហភាព/Temperature	☐☐.☐°C (digital)		ជីពចរ/Pulse	☐☐☐bpm
សំពាធឈាម/Blood Pressure	☐☐☐ / ☐☐ mm Hg		កំហាប់អុកស៊ីសែន/SaO2	I__I__I__I %

Haemorrhagic Manifestations

Dry Bleeding			
Petechiae	❑$_0$ No	❑$_1$ Yes	
Purpura or ecchymosis	❑$_0$ No	❑$_1$ Yes	
Wet Bleeding			
Bleeding nose (epistaxis)	❑$_0$ No	❑$_1$ Moderate	❑$_2$ Severe
Bleeding gums	❑$_0$ No	❑$_1$ Moderate	❑$_2$ Severe
Hematemesis, melena	❑$_0$ No	❑$_1$ Moderate	❑$_2$ Severe
Hematuria	❑$_0$ No	❑$_1$ Moderate	❑$_2$ Severe
Vaginal bleeding	❑$_0$ No	❑$_1$ Moderate	❑$_2$ Severe
Tourniquet test	❑$_0$Negative	❑$_1$ Positive	❑$_9$ Not Done

Warning Signs

Abdominal tenderness	❑$_0$ No	❑$_1$ Yes
Persistent vomiting	❑$_0$ No	❑$_1$ Yes
Lethargy/restlessness	❑$_0$ No	❑$_1$ Yes
Liver enlargement > 2 cm	❑$_0$ No	❑$_1$ Yes

Plasma Leakage

Ascites	❑$_0$ No	❑$_1$ Yes
Pleural effusion	❑$_0$ No	❑$_1$ Yes
Facial Oedema	❑$_0$ No	❑$_1$ Yes

Shock Signs

Cold / clammy extremities	❑$_0$ No	❑$_1$ Yes
Hypotension, weak/undetectable pulse	❑$_0$ No	❑$_1$ Yes
Delayed capillary refill (> than 3 seconds)	❑$_0$ No	❑$_1$ Yes
Narrow pulse pressure ($\leq$ 20 mm Hg)	❑$_0$ No	❑$_1$ Yes

Other Complications

Coma / Impaired Consciousness	❑$_0$ No	❑$_1$ Yes
Heart Failure	❑$_0$ No	❑$_1$ Yes
Myocardiopathy	❑$_0$ No	❑$_1$ Yes
Respiratory Distress	❑$_0$ No	❑$_1$ Yes
Encephalitis, Encephalopathy	❑$_0$ No	❑$_1$ Yes
Acute Liver Failure	❑$_0$ No	❑$_1$ Yes
Acute Renal Failure	❑$_0$ No	❑$_1$ Yes

Treatment on Admission (Day I)

Corticosteroid	☐$_0$ No	☐$_1$ Yes		Paracetamol	☐$_0$ No	☐$_1$ Yes
NSAID	☐$_0$ No	☐$_1$ Yes		Antibiotic	☐$_0$ No	☐$_1$ Yes
Aspirin	☐$_0$ No	☐$_1$ Yes				

Lactate Ringer's	☐$_0$ No	☐$_1$ Yes	☐☐☐☐	mL total / 24h
Acetate Ringer's	☐$_0$ No	☐$_1$ Yes	☐☐☐☐	mL total / 24h
5% Dextrose Lactate Ringer's	☐$_0$ No	☐$_1$ Yes	☐☐☐☐	mL total / 24h
5% Dextrose Acetate Ringer's	☐$_0$ No	☐$_1$ Yes	☐☐☐☐	mL total / 24h
5% Dextrose Normal Saline Sol	☐$_0$ No	☐$_1$ Yes	☐☐☐☐	mL total / 24h
Dextran 40 Plasma Expander	☐$_0$ No	☐$_1$ Yes	☐☐☐☐	mL total / 24h
Blood Transfusion	☐$_0$ No	☐$_1$ Yes	☐☐☐☐	mL
Packed Cells	☐$_0$ No	☐$_1$ Yes	☐☐☐☐	mL

សារជាតិទឹកដែលយើងផឹកតាមមាត់/**Oral Intake**	☐☐☐☐	mL total / 24h
ចំណុះទឹកនោមដែលហូរចេញ /**Urine Outcome**	☐☐☐☐	mL total / 24h

Investigations and Lab Testing on Admission (Day I)

Haematology

Platelets	☐$_0$ No	☐$_1$ Yes	☐☐☐	(x 10^9/L)
Leukocytes	☐$_0$ No	☐$_1$ Yes	☐☐.☐	(x 10^9/L)
Lymphocytes	☐$_0$ No	☐$_1$ Yes	☐☐.☐	(x 10^9/L)
Neutrophils	☐$_0$ No	☐$_1$ Yes	☐☐.☐	(x 10^9/L)
Hematocrit	☐$_0$ No	☐$_1$ Yes	☐☐	(x 10^9/L)
Hemoglobin	☐$_0$ No	☐$_1$ Yes	☐☐.☐	(%)
MCV	☐$_0$ No	☐$_1$ Yes	☐☐	(g/dL)
MCH	☐$_0$ No	☐$_1$ Yes	☐☐	(fl)

Chemistry

AST	☐$_0$ No	☐$_1$ Yes	☐☐☐☐	(UI/L)
ALT	☐$_0$ No	☐$_1$ Yes	☐☐☐☐	(UI/L)
Protein	☐$_0$ No	☐$_1$ Yes	☐☐	(g/L)

Ultrasound

	☐$_1$ Normal	☐$_2$ Abnormal	☐$_9$ Not Done	
If abnormal	☐$_1$ Ascitis	☐$_2$ Hepatomegaly	☐$_3$ Pleural effusion	☐$_4$ Other

Subjects Initials I__I__I__I **Subject ID Number I__I__I__I__I**

	Day II	Day III	Day IV	Day V
Date	□□ / □□	□□ / □□	□□ / □□	□□ / □□
Day of fever	□□	□□	□□	□□
General status	❑$_1$ Well ❑$_2$ Unwell	❑$_1$ Well ❑$_2$ Unwell	❑$_1$ Well ❑$_2$ Unwell	❑$_1$ Well ❑$_2$ Unwell
Temperature (^{0}C)	□□.□	□□.□	□□.□	□□.□
Pulse (bpm)	□□□	□□□	□□□	□□□
Systolic BP	□□□	□□□	□□□	□□□
Diastolic BP	□□□	□□□	□□□	□□□
SaO2 % (%)	□□□	□□□	□□□	□□□
**Hemorrhagic Manifestations**				
Petechiae	❑$_0$ N ❑$_1$ Y	❑$_0$ N ❑$_1$ Y	❑$_0$ N ❑$_1$ Y	❑$_0$ N ❑$_1$ Y
Purpura / ecchymosis	❑$_0$ N ❑$_1$ Y	❑$_0$ N ❑$_1$ Y	❑$_0$ N ❑$_1$ Y	❑$_0$ N ❑$_1$ Y
Bleeding nose / gums	❑$_0$ N ❑$_1$ Y	❑$_0$ N ❑$_1$ Y	❑$_0$ N ❑$_1$ Y	❑$_0$ N ❑$_1$ Y
Hematemesis / melena	❑$_0$ N ❑$_1$ Y	❑$_0$ N ❑$_1$ Y	❑$_0$ N ❑$_1$ Y	❑$_0$ N ❑$_1$ Y
Vaginal bleeding	❑$_0$ N ❑$_1$ Y	❑$_0$ N ❑$_1$ Y	❑$_0$ N ❑$_1$ Y	❑$_0$ N ❑$_1$ Y
Tourniquet Test	□	□	□	□
	(Code "0"Negative, "1" for positive, "9" for not performed)			
**Warning Signs**				
Abdominal tenderness	❑$_0$ N ❑$_1$ Y	❑$_0$ N ❑$_1$ Y	❑$_0$ N ❑$_1$ Y	❑$_0$ N ❑$_1$ Y
Persistent vomiting	❑$_0$ N ❑$_1$ Y	❑$_0$ N ❑$_1$ Y	❑$_0$ N ❑$_1$ Y	❑$_0$ N ❑$_1$ Y
Lethargy/ restlessness	❑$_0$ N ❑$_1$ Y	❑$_0$ N ❑$_1$ Y	❑$_0$ N ❑$_1$ Y	❑$_0$ N ❑$_1$ Y
Liver enlargement	❑$_0$ N ❑$_1$ Y	❑$_0$ N ❑$_1$ Y	❑$_0$ N ❑$_1$ Y	❑$_0$ N ❑$_1$ Y
**Plasma Leakage Signs**				
Ascites	❑$_0$ N ❑$_1$ Y	❑$_0$ N ❑$_1$ Y	❑$_0$ N ❑$_1$ Y	❑$_0$ N ❑$_1$ Y
Pleural effusion	❑$_0$ N ❑$_1$ Y	❑$_0$ N ❑$_1$ Y	❑$_0$ N ❑$_1$ Y	❑$_0$ N ❑$_1$ Y
Facial edema	❑$_0$ N ❑$_1$ Y	❑$_0$ N ❑$_1$ Y	❑$_0$ N ❑$_1$ Y	❑$_0$ N ❑$_1$ Y
Ultrasound	□	□	□	□
	(Code "1"for normal, "2" for abnormal, "9" for not performed)			
If abnormal, specify	□	□	□	□
	(Code "1"for ascites, "2" for hepatomegaly, "3" for pleural effusion, and "4" for other)			
**Shocks Signs**				
Cold / clammy extremities	❑$_0$ N ❑$_1$ Y	❑$_0$ N ❑$_1$ Y	❑$_0$ N ❑$_1$ Y	❑$_0$ N ❑$_1$ Y
Hypotension, weak or undetectable pulse	❑$_0$ N ❑$_1$ Y	❑$_0$ N ❑$_1$ Y	❑$_0$ N ❑$_1$ Y	❑$_0$ N ❑$_1$ Y
Delayed capillary refill	❑$_0$ N ❑$_1$ Y	❑$_0$ N ❑$_1$ Y	❑$_0$ N ❑$_1$ Y	❑$_0$ N ❑$_1$ Y
Narrow pulse pressure	❑$_0$ N ❑$_1$ Y	❑$_0$ N ❑$_1$ Y	❑$_0$ N ❑$_1$ Y	❑$_0$ N ❑$_1$ Y
Coma / Impaired Consciousness	❑$_0$ N ❑$_1$ Y	❑$_0$ N ❑$_1$ Y	❑$_0$ N ❑$_1$ Y	❑$_0$ N ❑$_1$ Y
Other Severe Organ Involvement	❑$_0$ N ❑$_1$ Y	❑$_0$ N ❑$_1$ Y	❑$_0$ N ❑$_1$ Y	❑$_0$ N ❑$_1$ Y

Subjects Initials I__I__I__I   Subject ID Number I__I__I__I__I__I

	Day I__I	Day I__I	Day I__I	Day I__I
Date	☐☐/☐☐	☐☐/☐☐	☐☐/☐☐	☐☐/☐☐
**Medications**				
Corticosteroid	☐$_0$N ☐$_1$Y	☐$_0$N ☐$_1$Y	☐$_0$N ☐$_1$Y	☐$_0$N ☐$_1$Y
NSAID	☐$_0$N ☐$_1$Y	☐$_0$N ☐$_1$Y	☐$_0$N ☐$_1$Y	☐$_0$N ☐$_1$Y
Aspirin	☐$_0$N ☐$_1$Y	☐$_0$N ☐$_1$Y	☐$_0$N ☐$_1$Y	☐$_0$N ☐$_1$Y
Paracetamol	☐$_0$N ☐$_1$Y	☐$_0$N ☐$_1$Y	☐$_0$N ☐$_1$Y	☐$_0$N ☐$_1$Y
Antibiotic	☐$_0$N ☐$_1$Y	☐$_0$N ☐$_1$Y	☐$_0$N ☐$_1$Y	☐$_0$N ☐$_1$Y
**Fluid infusion**				
Lactate Ringer's	☐N ☐Y ☐☐☐☐mL	☐N ☐Y ☐☐☐☐mL	☐N ☐Y ☐☐☐☐mL	☐N ☐Y ☐☐☐☐mL
Acetate Ringer's	☐N ☐Y ☐☐☐☐mL	☐N ☐Y ☐☐☐☐mL	☐N ☐Y ☐☐☐☐mL	☐N ☐Y ☐☐☐☐mL
D5 Ringer Lactate	☐N ☐Y ☐☐☐☐mL	☐N ☐Y ☐☐☐☐mL	☐N ☐Y ☐☐☐☐mL	☐N ☐Y ☐☐☐☐mL
D5 Ringer Acetate	☐N ☐Y ☐☐☐☐mL	☐N ☐Y ☐☐☐☐mL	☐N ☐Y ☐☐☐☐mL	☐N ☐Y ☐☐☐☐mL
D5 Normal Saline	☐N ☐Y ☐☐☐☐mL	☐N ☐Y ☐☐☐☐mL	☐N ☐Y ☐☐☐☐mL	☐N ☐Y ☐☐☐☐mL
Dextran 40	☐N ☐Y ☐☐☐☐mL	☐N ☐Y ☐☐☐☐mL	☐N ☐Y ☐☐☐☐mL	☐N ☐Y ☐☐☐☐mL
Blood transfusion	☐N ☐Y ☐mL	☐N ☐Y ☐mL	☐N ☐Y ☐mL	☐N ☐Y ☐mL
Packed cells	☐N ☐Y ☐mL	☐N ☐Y ☐mL	☐N ☐Y ☐mL	☐N ☐Y ☐mL
Oral Intake	☐☐☐☐mL	☐☐☐☐mL	☐☐☐☐mL	☐☐☐☐mL
Urine Outcome	☐☐☐☐mL	☐☐☐☐mL	☐☐☐☐mL	☐☐☐☐mL

**Laboratory (Compulsory)**					
Hematocrit ***(highest)***	☐☐	☐☐	☐☐	☐☐	In %
Time:	☐☐:☐☐	☐☐:☐☐	☐☐:☐☐	☐☐:☐☐	
Hematocrit ***(lowest)***	☐☐	☐☐	☐☐	☐☐	In %
Time:	☐☐:☐☐	☐☐:☐☐	☐☐:☐☐	☐☐:☐☐	
**Laboratory (Optional)**					
Platelets ***(lowest)***	☐☐☐	☐☐☐	☐☐☐	☐☐☐	($x10^9$/L)
Leukocytes ***(lowest)***	☐☐.☐	☐☐.☐	☐☐.☐	☐☐.☐	($x10^9$/L)
Lymphocytes ***(lowest)***	☐☐.☐	☐☐.☐	☐☐.☐	☐☐.☐	($x10^9$/L)
Neutrophils	☐☐.☐	☐☐.☐	☐☐.☐	☐☐.☐	($x10^9$/L)
Hemoglobin ***(lowest)***	☐☐.☐	☐☐.☐	☐☐.☐	☐☐.☐	(g/dl)
MCV	☐☐	☐☐	☐☐	☐☐	(fl)
MCH	☐☐	☐☐	☐☐	☐☐	(pg)
AST ***(highest)***	☐☐☐☐	☐☐☐☐	☐☐☐☐	☐☐☐☐	Units / L
ALT ***(highest)***	☐☐☐☐	☐☐☐☐	☐☐☐☐	☐☐☐☐	Units / L

**Assessment at Discharge**	Date: ☐☐ / ☐☐ / ☐☐ ☐☐

Compulsory Tests at Discharge

Blood Count ❑$_0$ Not Performed ❑$_1$ Performed

Platelets	☐☐☐	(x 10^9/L)	Hematrocrit	☐☐	(%)
Leukocytes	☐☐.☐	(x 10^9/L)	Hemoglobin	☐☐.☐	(g/dL)
Lymphocytes	☐☐.☐	(x 10^9/L)	MCV	☐☐	(fl)
Neutrophils	☐☐.☐	(x 10^9/L)	MCH	☐☐	(pg)

Clinical Outcome at Discharge

The patient has a clinical diagnosis of dengue ❑$_0$ No ❑$_1$ Yes

If the patient has a clinical diagnosis of dengue, what is the clinical outcome according to the **WHO 1997** Classification ***tick only 1 case)***

- ❑$_0$ Uncomplicated Dengue Fever (no DHF, no DSS)
- ❑$_1$ Dengue Hemorrhagic Fever, Grade I
- ❑$_2$ Dengue Hemorrhagic Fever, Grade II
- ❑$_3$ Dengue Hemorrhagic Fever, Grade III
  (*Grade III = "mild" DSS: bleeding with moderate circulatory failure)*
- ❑$_4$ Dengue Hemorrhagic Fever, Grade IV
  (Grade IV = "severe" DSS: bleeding with undetectable blood pressure)

If the patient has a clinical diagnosis of dengue, what is the clinical outcome according to the **WHO 2009** Classification ***tick only 1 case)***

- ❑$_1$ Dengue Fever WITHOUT any Waning Signs
- ❑$_2$ Dengue Fever WITH Warning Sign(s)
- ❑$_3$ Severe Dengue

The patient has a confirmed diagnosis of malaria ❑$_0$ No ❑$_1$ Yes

The patient has a clinical diagnosis of ARI / Flu ❑$_0$ No ❑$_1$ Yes

The patient has another clinical condition ❑$_0$ No ❑$_1$ Yes

If "yes", specify: ....

Status at Discharge

- ❑$_1$ Recovered
- ❑$_2$ Died
- ❑$_3$ Transferred
- ❑$_4$ Left against medical advice
