# Supplementary material for: Early diagnosis of dengue disease severity in a resource-limited Asian country
Source: BMC Infect Dis. 2016 Sep 26;16:512. doi: 10.1186/s12879-016-1849-8 (PMC5036306; doi:10.1186/s12879-016-1849-8)
Supplement: Additional file 3: — Supplementary Data Analyses. (DOC 190 kb) [file 12879_2016_1849_MOESM3_ESM.doc]

Early diagnosis and Predictors of Dengue Disease Severity, National Paediatric Hospital, Phnom Penh, 2011 – 2013

# *Supporting Information File 3*

A joint project between:

The Ministry of Health Cambodia,

The National Dengue Control Programme, the National Paediatric Hospital (Phnom Penh),

Sanofi Pasteur, the National University of Singapore, Institut Pasteur in Cambodia,

Tan Tock Seng Hospital / Communicable Disease Centre Singapore, and the

Regional Emerging Diseases Intervention (REDI) Centre, Singapore

Table of Contents

**Appendix 1:** Inclusion flow …………………………………………………………………………………………………...................3

**Appendix 2:** Clinical characteristics of laboratory-positive and laboratory-negative patients.................4

- **Table 1:** - Symptoms on admission
- **Table 2:** Haemorrhagic manifestations and plasma leakage signs on admission
- **Table 3:** Shock and warning signs on admission
- **Table 4:** Severity criteria on admission
- **Table 5:** Laboratory parameters on admission

**Appendix 3:** Determinants for dengue infection (multivariate analysis)…………………………………...............8

- **Table 6:** Independent predictors associated with laboratory-confirmation of dengue Infection (results of liver transaminases dosage included in the model)
- **Table 7:** Independent predictors associated with laboratory-confirmation of dengue Infection (results of liver transaminases dosage not included in the model)

**Appendix 1 – Inclusion Flow**

**
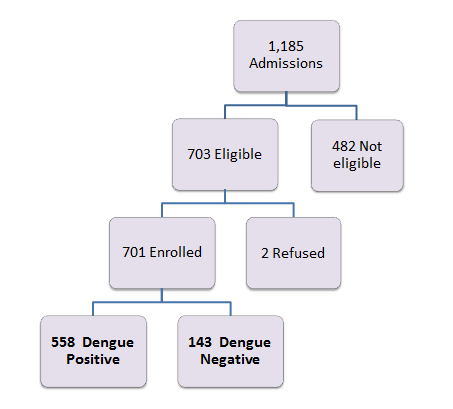
**

**Appendix 2**

| Table 1 - Symptoms on admission of laboratory-positive and laboratory-negative patients, Dengue Surveillance Study (N = 701), National Paediatric Hospital, Phnom Penh, Sep. 2011 - Jan. 2013 | | | | | |
| --- | --- | --- | --- | --- | --- |
|  |  | Neg.-Denv.  n = 143 | Pos.-Denv.  n = 558 | P - value | OR (95% CI) |
| **Symptoms on admission** | |  |  |  |  |
|  | Rash | 17.5 | 27.8 | 0.012 | 1.81 (1.13 - 2.90) |
|  | Pallor or cool skin | 2.8 | 2.9 | 1.00 | 1.03 (0.34 - 3.11) |
|  | Headache | 58.0 | 62.2 | 0.36 | 1.19 (0.81 - 1.72) |
|  | Retro-orbital pain | 16.`1 | 28.7 | 0.002 | 2.09 (1.29 - 3.39) |
|  | Myalgia | 22.4 | 37.5 | 0.001 | 2.08 (1.35 - 3.19) |
|  | Arthralgia | 25.4 | 30.5 | 0.23 | 1.29 (0.85 - 1.96) |
|  | Myalgia or arthralgia | 31.0 | 42.3 | 0.014 | 1.63 (1.10 - 2.41) |
|  | Anorexia | 53.8 | 62.9 | 0.048 | 1.45 (1.00 - 2.11) |
|  | Nausea | 16.8 | 22.8 | 0.12 | 1.46 (0.90 - 2.36) |
|  | Vomiting | 62.9 | 78.9 | < 0.001 | 2.19 (1.47 - 3.26) |
|  | Nausea or vomiting | 69.2 | 82.3 | 0.001 | 2.06 (1.35 - 3.12) |
|  | Abdominal pain | 74.1 | 85.3 | 0.001 | 2.02 (1.30 - 3.15) |
|  | Abdominal distension | 1.4 | 2.7 | 0.54 | 1.94 (0.44 - 8.61) |
|  | Diarrhoea | 5.6 | 3.9 | 0.38 | 0.69 (0.30 - 1.59) |
|  | Jaundice | 0.0 | 0.2 | 1.00 | N.A. |
|  | Restlessness | 28.9 | 36.1 | 0.16 | 1.33 (0.89 - 1.99) |
|  | Lethargy | 19.7 | 25.1 | 0.18 | 1.36 (0.86 - 2.15) |
|  | Lethargy or restlessness | 48.6 | 56.8 | 0.029 | 1.51 (1.04 - 2.18) |
|  | Sore throat | 14.1 | 6.3 | 0.002 | 0.41 (0.23 - 0.73) |
|  | Cough | 33.8 | 12.9 | < 0.001 | 0.29 (0.19 - 0.44) |
|  | Breathlessness | 2.1 | 0.7 | 0.15 | 0.33 (0.07 - 1.51) |
|  | Cough + breathlessness | 34.5 | 13.1 | < 0.001 | 0.29 (0.19 - 0.44) |
|  | Cough + breathlessness + sore throat | 40.8 | 17.6 | < 0.001 | 0.31 (0.21 - 0.46) |

| Table 2 –Haemorrhagic manifestations and plasma leakage on admission of laboratory-positive and laboratory-negative patients, Dengue Surveillance Study (N = 701), National Paediatric Hospital, Phnom Penh, Sep. 2011 - Jan. 2013 (part 1) | | | | | |
| --- | --- | --- | --- | --- | --- |
|  |  | Neg.-Denv.  n = 143 | Pos.-Denv.  n = 558 | P - value | OR (95% CI) |
| **Haemorrhagic Manifestations (%)** | |  |  |  |  |
|  | Petechiae | 9.8 | 20.7 | 0.003 | 2.40 (1.33 - 4.32) |
|  | Purpura / ecchymosis | 8.4 | 12.7 | 0.090 | 1.72 (0.91 - 3.27) |
|  | ***Any dry bleeding*** | 15.4 | 29.7 | 0.001 | 2.34 (1.42 - 3.80) |
|  | Bleeding nose | 2.1 | 4.0 | 0.29 | 1.92 (0.56 - 6.51) |
|  | Bleeding gums | 0.0 | 2.3 | 0.082 | N.A. |
|  | Hematuria | N.A. | N.A. | N.A | N.A. |
|  | Vaginal bleeding | 0.7 | 0.4 | 0.049 | N.A. |
|  | Hematemesis, melena | 4.2 | 5.2 | 0,62 | 1.25 (0.51 - 3.1) |
|  | ***Any wet (mucosal) bleeding*** | 7.0 | 10.4 | 0.22 | 1.54 (0.77 - 3.11) |
|  | ***Any external bleeding (wet or dry bleeding)*** | 20.3 | 35.6 | < 0.001 | 2.17 (1.39 - 3.38) |
|  | Positive tourniquet test (TT) (N = 663) | 48.9 | 74.3 | < 0.001 | 3.02 (2.05 - 4.46) |
|  | ***Any haemorrhagic manifestations (with TT)*** | 58.7 | 79.4 | < 0.001 | 2.71 (1.83 - 4.00) |
| **Plasma Leakage (%)** | |  |  |  |  |
|  | Clinical ascites | 1.4 | 8.8 | 0.002 | 6.81 (1.64 - 28.36) |
|  | Clinical pleural effusion | 2.1 | 13.3 | < 0.001 | 7.16 (2.22 - 23.01) |
|  | ***Clinical fluid accumulation**** | 2.8 | 14.6 | < 0.001 | 5.93 (2.13 - 16.46) |
|  | Elevated haematocrit (> 20%) for age** | 0.7 | 6.3 | 0.007 | 9.59 (1.30 - 70.6) |
|  | ***Any plasma leakage sign (3 criteria)*** | 3.5 | 17.6 | < 0.001 | 5.88 (2.35 - 14.73) |
|  | Elevated haematocrit (> 20%) from baseline*** | 6.3 | 18.1 | 0.010 | 3.27 (1.29 - 8.43) |
|  | ***Any plasma leakage sign (4 criteria)*** | 7.0 | 23.3 | < 0.001 | 4.04 (2.06 - 7.91) |
| *( * ) - Either clinical ascites or pleural effusion*  *(**) - Haematocrit value assessed on admission compared to reference tables for age and gender*  *(**) - Haematocrit value assessed on admission compared to haematocrit assesses at discharge* | | | | | |

| Table 3 - Shock and warning signs on admission of laboratory-positive and laboratory-negative patients, Dengue Surveillance Study (N = 701), National Paediatric Hospital, Phnom Penh, Sep. 2011 - Jan. 2013 | | | | | |
| --- | --- | --- | --- | --- | --- |
|  |  | Neg.-Denv.  n = 143 | Pos.-Denv.  n = 558 | P - value | OR (95% CI) |
| **Signs of cardio-vascular shock (%)** | |  |  |  |  |
|  | _1_Cold / clammy extremity | 0.0 | 7.7 | 0.001 | N.A. |
|  | _2_Weak / undetectable pulse | 0.0 | 4.9 | 0.007 | N.A. |
|  | _3_Delayed capillary refill (> 3 seconds) | 0.7 | 5.4 | 0.015 | 8.09 (1.09 - 59.90) |
|  | _4_Narrow pulse pressure (by clinician) | 0.0 | 3.2 | 0.033 | N.A. |
|  | ***Any shock signs above (any-4)*** | 0.7 | 8.1 | 0.001 | 12.5 (1.7 - 91.51) |
|  | _4a_Narrow pulse pressure (computed from CRF*) | 2.1 | 3.6 | 0.378 | 1.72 (0.51 - 5.89) |
|  | _5_Low blood pressure for age** | 7.1 | 11.6 | 0.12 | 1.71 (0.86 - 3.43) |
|  | ***Any shock signs (****any-5: 1, 2, 3, 4 or 5****)*** | 7.8 | 18.6 | 0.002 | 2.70 (1.41 - 5.19) |
| **Warning signs (%)** | |  |  |  |  |
|  | Abdominal tenderness | 11.9 | 26.4 | < 0.001 | 2.66 (1.55 - 4.57) |
|  | Persistent vomiting | 0.7 | 6.1 | 0.008 | 9.42 (1.25 - 68.15) |
|  | Clinical fluid accumulation | 2.8 | 14.6 | < 0.001 | 5.93 (2.13 - 16.46) |
|  | Mucosal bleeding | 7.0 | 10.4 | 0.22 | 1.54 (0.77 - 3.11) |
|  | Lethargy / restlessness | 2.1 | 21.4 | < 0.001 | 12.7 (3.97 - 40.59) |
|  | Liver enlargement > 2 cm | 5.6 | 24.5 | < 0.001 | 5.64 (2.61 11.44) |
|  | Presence on warning signs |  |  |  |  |
|  | - at least one | 23.1 | 55.7 | < 0.001 | 4.19 (2.78 - 6.41) |
|  | - at least two | 5.6 | 26.7 | < 0.001 | 6.14 (2.94 - 12.85) |
|  | - at least three | 1.4 | 12.9 | < 0.001 | 10.44 (2.53 - 43.10) |
|  | - at least four | 0 | 5.2 | 0.005 | N.A. |
|  | - at least five | 0 | 2.2 | 0.14 | N.A |
|  | - All are present | 0 | 0.4 | 1.00 | N.A |
| *( * ) - Narrow pulse pressure (computed for CRF data): if Systolic BP - Diastolic BP < 20 mm Hg*  *(**) - Hypotension for age (computed from CRF data): if systolic BP < 80 mm hg (for < 5 years old) and < 90 mm Hg (for > 5 years old)* | | | | | |

| Table 4 - Severity criteria on admission of laboratory-positive and laboratory-negative patients, Dengue Surveillance Study (N = 701), National Paediatric Hospital, Phnom Penh, Sep. 2011 - Jan. 2013 | | | | | |
| --- | --- | --- | --- | --- | --- |
|  |  | Neg.-Denv.  n = 143 | Pos.-Denv.  n = 558 | P - value | OR (95% CI) |
| **Severe plasma leakage** | |  |  |  |  |
|  | Fluid accumulation with shock | 0.7 | 6.8 | 0.004 | 10.33 (1.41 - 76.23) |
| **Severe bleeding** | |  |  |  |  |
|  | Any significant bleeding | 0.0 | 0.2 | 1.00 | N.A. |
| **Impaired consciousness** | |  |  |  |  |
|  | Coma / convulsion | 0.0 | 0.4 | 1.00 | N.A |
| **Organ impairment** | |  |  |  |  |
|  | Heart failure | 0.7 | 0.5 | 1.00 | 0.77 (0.08 - 7.46) |
|  | Myocardiopathy (N = 455) | 0.0 | 0.0 | N.A. | N.A |
|  | Respiratory distress (N = 455) | 0.0 | 0.3 | 1.00 | N.A. |
|  | Encephalitis, encephalopathy (N = 455) | 0.0 | 0.3 | 1.00 | N.A |
|  | Acute liver failure (N = 455) | 0.0 | 0.0 | N.A. | N.A |
|  | Liver AST or ALT > 1000 | 0.0 | 0.2 | 1.00 | N.A |
|  | Acute renal failure (N = 455) | 0.0 | 0.0 | N.A. | N.A |
|  | Any Organ Impairment Criteria | 0.7 | 1.1 | 1.00 | 1.54 (0.18 - 12.92) |
| **Any severity criteria** | | **1.4** | **7.9** | **0.005** | **6.03 (1.44 - 25.20)** |
| *( * ) - Narrow pulse pressure (computed for CRF data): if Systolic BP - Diastolic BP < 20 mm Hg*  *(**) - Hypotension for age (computed from CRF data): if systolic BP < 80 mm hg (for < 5 years old) and < 90 mm Hg (for > 5 years old)* | | | | | |

| Table 5 - Laboratory parameters on admission of laboratory-positive and laboratory-negative patients, Dengue Surveillance Study (N = 701), National Paediatric Hospital, Phnom Penh, Sep. 2011 - Jan. 2013 | | | | | |
| --- | --- | --- | --- | --- | --- |
|  |  | Neg.-Denv.  n = 143 | Pos.-Denv.  n = 558 | P - value | OR (95% CI) |
| Leukocyte count (N = 694) | |  |  |  |  |
|  | Median [IQR] | 5.6 [4.0 - 8.4] | 4.6 [3.2 - 6.6] | < 0.001 | N.A. |
|  | < 4,000 / mm^3^ | 24.5 | 41.0 | < 0.001 | 2.14 (1.41 - 3.25) |
| Neutrophil count (N = 503) | |  |  |  |  |
|  | Median [IQR] | 3.1 [1.8 - 5.3] | 2.2 [1.5 - 3.4] | < 0.001 | N.A. |
|  | < 3,000 / mm^3^ | 38.5 | 44.1 | 0.22 | 1.26 (0.87 - 1.83) |
| Lymphocyte count (N = 692) | |  |  |  |  |
|  | Median [IQR] | 1.7 [1.3 - 2.9] | 1.6 [1.1 - 2.5] | 0.029 | N.A. |
|  | < 1,500 / mm^3^ | 38.5 | 44.1 | 0.22 | 1.26 (0.87 - 1.83) |
| Platelet count (N = 696) | |  |  |  |  |
|  | Median [IQR] | 141 [96 - 181] | 87 [[54 - 125] | < 0.001 | N.A. |
|  | < 20,000 / mm^3^ | 2.1 | 2.5 | 0.76 | 1.21 (0.34 - 4.27) |
|  | < 50,000 / mm^3^ | 6.3 | 22.2 | < 0.001 | 4.25 (2.10 - 8.60) |
|  | < 100,000 / mm^3^ | 25.9 | 58.8 | < 0.001 | 4.08 (2.71 - 6.16) |
| Haemoglobin (N = 505) | |  |  |  |  |
|  | Median | 12.1 [11.3 - 13.0] | 12.9 [12.0 - 14.0] | < 0.001 | N.A. |
| Haematocrit (N = 696) | |  |  |  |  |
|  | Median [IQR] | 37 [35 - 40] | 40 [37 - 43] | < 0.001 | N.A. |
|  | > 45% | 7.7 | 18.4 | 0.002 | 2.71 (1.41 - 5.21) |
| ALT (N = 558) | |  |  |  |  |
|  | Median [IQR] | 55 [37.75 - 90.5] | 76 [49 - 120] | < 0.001 | N.A. |
|  | > 40 U / mL | 62.7 | 81.0 | < 0.001 | 2.53 (1.60 - 3.98) |
|  | > 60 U / mL | 38.2 | 58.2 | < 0.001 | 2.25 (1.47 - 3.45) |
|  | > 100 U / mL | 13.6 | 29.8 | 0.001 | 2.68 (1.5 - 4.79) |
| AST (n = 557) | |  |  |  |  |
|  | Median [IQR] | 48 [30 - 77] | 70 [46 - 110] | < 0.001 | N.A. |
|  | > 40 U / mL | 70.0 | 83.5 | 0.001 | 2.16 (1.34 - 3.49) |
|  | > 60 U / mL | 43.6 | 64.5 | < 0.001 | 2.34 (1.53 - 3.59] |
|  | > 100 U / mL | 15.5 | 34.8 | < 0.001 | 2.92 (1.68 - 5.07) |

**Appendix 3**

| Table 6 - Independent predictors associated with laboratory-confirmation of dengue Infection, Dengue Surveillance Study, National Paediatric Hospital, Phnom Penh, Sep. 2011 - Jan. 2013 (model including the results of liver transaminases dosage) | | | | | |
| --- | --- | --- | --- | --- | --- |
| **MODEL A** | Crude OR  (95% CI) |  | Adjusted OR  (95% CI) | P-value | *Label* |
| Age > 5 years | 1.62 (1.04 - 2.52) |  | 1,01 (0.60 - 2.13) | 0.75 | *AgM5* |
| Rash | 1.81 (1.13 - 2.90) |  | 1.17 (0.65 - 2.08) | 0.59 | *P1240* |
| Retro-orbital pain | 2.09 (1.29 - 3.39) |  | 1.33 (0.73 - 2.44) | 0.35 | *P1290* |
| Myalgia / arthralgia | 1.63 (1.10 - 2.41) |  | 0.95 (0.57 - 1.61) | 0.87 | *MAB* |
| **Nausea / vomiting** | 2.06 (1.35 - 3.12) |  | **1.71 (0.96 - 3.050** | **0.070** | *NVB* |
| Lethargy / restlessness | 1.51 (1.04 - 2.18) |  | 1.21 (0.74 - 1.99) | 0.43 | *LRB* |
| **Absence of URI symptoms*** | 3.23 (2.16 - 4.82) |  | **2.53 (1.47 - 4.37)** | **0.001** | *NURI* |
| **Positive tourniquet test** | 3.04 (2.02 - 4.57) |  | **2.17 (1.32 - 3.59)** | **0.002** | *TT* |
| **Liver enlargement** | 5.64 (2.61 - 11.44) |  | **2.41 (0.95 - 6,11)** | **0.063** | *a1230* |
| Plasma leakage sign | 5.94 (2.14-16.49) |  | 2.41 (0.65 - 8.87) | 0.19 | *APFB* |
| **Leukocytes < 4,000** | 2.14 (1.41 - 3.25) |  | **3.60 (2.04 - 6.37)** | **< 0.001** | *L4000* |
| **Platelet < 100,000** | 4.08 (2.71 - 6.16) |  | **2.28 (1.32 - 6.37)** | **0.003** | *P100* |
| Haematocrit > 45% | 2.71 (1.41 - 5.21) |  | 1.32 (0.58 - 3.91) | 0.50 | *H45* |
| **AST > 60 U / mL** | 2.25 (1.47 - 3.45) |  | **2.31 (1.42 - 3.75)** | **0.001** | *AST60* |
| ** URI = Upper Respiratory Tract Infection*  *Multivariate logistic regression analysis conducted among 530 patients (missing information in 171 cases)* | | | | | |

| Table 7 - Independent predictors associated with laboratory-confirmation of dengue Infection, Dengue Surveillance Study, National Paediatric Hospital, Phnom Penh, Sep. 2011 - Jan. 2013 (model **NOT** including the results of liver transaminases dosage) | | | | | |
| --- | --- | --- | --- | --- | --- |
| **MODEL B** | Crude OR  (95% CI) |  | Adjusted OR  (95% CI) | P-value | *Label* |
| Age > 5 years | 1.62 (1.04 - 2.52) |  | 1,11 (0.66 - 1.89) | 0.69 | *AgM5* |
| Rash | 1.81 (1.13 - 2.90) |  | 1.37 (0.80 - 2.36) | 0.26 | *P1240* |
| Retro-orbital pain | 2.09 (1.29 - 3.39) |  | 1.40 (0.79 - 2.47) | 0.25 | *P1290* |
| Myalgia / arthralgia | 1.63 (1.10 - 2.41) |  | 1.02 (0.64 - 1.63) | 0.95 | *MAB* |
| **Nausea / vomiting** | 2.06 (1.35 - 3.12) |  | **1.81 (1.10 - 2.98)** | **0.020** | *NVB* |
| Lethargy / restlessness | 1.51 (1.04 - 2.18) |  | 1.32 (0.85 - 2.03) | 0.21 | *LRB* |
| **Absence of URI symptoms** | 3.23 (2.16 - 4.82) |  | **2.59 (1.63 - 4.14)** | **< 0.001** | *NURI* |
| **Positive tourniquet test** | 3.04 (2.02 - 4.57) |  | **2.22 (1.43 - 3.44)** | **< 0.001** | *TT* |
| **Liver enlargement** | **5.64 (2.61 - 11.44)** |  | **2.97 (1.31 - 6.74)** | **0.009** | *a1230* |
| Plasma leakage sign | 5.94 (2.14-16.49) |  | 1.85 (0.61 - 5.70) | 0.28 | *APFB* |
| **Leukocytes < 4,000** | 2.14 (1.41 - 3.25) |  | **2.96 (1.82 - 4.83)** | **< 0.001** | *L4000* |
| **Platelet < 100,000** | 4.08 (2.71 - 6.16) |  | **2.37 (1.48 - 3.82)** | **< 0.001** | *P100* |
| Haematocrit > 45% | 2.71 (1.41 - 5.21) |  | 1.35 (0.64 - 2.86) | 0.44 | *H45* |
| *Multivariate logistic regression analysis conducted among 653 patients (missing information in 48 cases)* | | | | | |
